# Supporting Effective Transitions From University to Post-graduation for Autistic Students

**DOI:** 10.3389/fpsyg.2021.768429

**Published:** 2022-02-07

**Authors:** Rebecca Lucas, Eilidh Cage, Alana I. James

**Affiliations:** ^1^School of Psychology, University of Roehampton, London, United Kingdom; ^2^Department of Psychology, Faculty of Natural Sciences, University of Stirling, Stirling, United Kingdom; ^3^School of Psychology and Clinical Language Sciences, University of Reading, Reading, United Kingdom

**Keywords:** autism, transition, graduates, university, higher education, employment

## Abstract

**Background:**

The number of autistic students graduating is increasing; however, little is known regarding their transition out of university. Understanding this transition is particularly pertinent with regard to the employment of autistic graduates. It is vital that we understand autistic people’s experiences of the transition and identify what support would be beneficial during this time.

**Method:**

Thirty-four autistic graduates from the United Kingdom took part in a mixed-methods study exploring their transition experience. Both quantitative and qualitative questions were used to obtain in-depth information concerning participants’ experiences. Participants completed questions regarding their experiences and emotions in relation to the transition, the support they received for the transition, and their career and post-graduation plans.

**Results:**

Participants reported high levels of fear and low preparedness for the transition. They did not feel well supported in preparing for the transition or for their future career. In the 6 months pre-graduation, 59% of participants had accessed emotion-related transition support and 70% accessed career-related support. Post-graduation, one-third accessed emotion-related or career-related support. Perspectives on this accessed support were mixed, as were transition experiences. Additional support desired included preparation for life changes, career planning, employment accessibility, and autism-specific support. Advice for future students centered on forward planning.

**Conclusion:**

These results highlight the importance of supporting autistic students with the transition out of university. Service provision should be tailored to autistic students’ needs and support early planning for the transition.

## Introduction

Increasing numbers of autistic individuals are attending university: in the 2019/20 academic year, 5,785 first-year UK-domiciled undergraduate students declared a diagnosis of autism (Higher Education Statistics Agency [HESA], 2021), a fourfold increase on the 1,065 students who did so in 2009/2010 (Higher Education Statistics Agency [HESA], 2011). Although autistic students^1^ may be at higher risk of not continuing their studies (Cage and Howes, 2020), many do successfully graduate (Anderson et al., 2017; Richardson, 2017). However, securing and maintaining subsequent employment can be a challenge. Six months post-graduation, 12.2% of 2018 autistic graduates were unemployed compared to 5.1% of non-disabled graduates (Association of Graduate Careers Advisory Services [AGCAS] Disability Task Group, 2021). Further, while 60.4% of United Kingdom 2018 graduates without a disability were in full-time work 6 months post-graduation, this was only the case for 36.4% of autistic graduates (Association of Graduate Careers Advisory Services [AGCAS] Disability Task Group, 2021).

Autistic people bring numerous skills and strengths to workplaces (Bury et al., 2020); however, the previously mentioned statistics indicate that autistic students may benefit from additional support for the transition out of university and into employment. Currently, many autistic students make this transition either without any support or only with support from services that are available to the whole student population (van Schalkwyk and Volkmar, 2017). It has been recognized that professional services within the university environment could better support *all* students with this transition (National Educational Association of Disabled Students, 2012). To target support effectively, a greater understanding of the challenges that autistic students specifically face is needed, as this study set out to explore.

### University to Post-graduation Transition

The transition from university is a pivotal time in any student’s life, marked by great change. This period may be especially challenging for autistic students. Autistic individuals can have preferences for structure, routine, and familiarity (American Psychological Association [APA], 2013), which can link to intolerance of uncertainty and mean change can be challenging (Maisel et al., 2016). The transition out of university involves leaving university and entering employment, further study, or a period of unemployment. Thus, the end of undergraduate studies is characterized by departure from routine and loss of support networks. Adaption to new environments will be necessary if the graduate moves location and/or enters a new place of study or work. Encountering new physical environments and meeting unfamiliar people can be anxiety provoking for many autistic people (Van Hees et al., 2015). Difficulties may be exacerbated if the individual needs to acclimatize to the sensory environment (Robertson and Simmons, 2015).

Mental health difficulties may further impact this transition. Around 70% of autistic individuals have co-occurring mental health conditions, most frequently anxiety and depression (Mazefsky et al., 2008; Simonoff et al., 2008; Skokauskas and Gallagher, 2010; van Steensel et al., 2013). It is unknown whether the transition out of university exacerbates mental health conditions for autistic graduates, or whether it contributes to the development of new mental health difficulties. However, even for non-autistic students, mental health conditions are associated with poorer experiences of transition and higher rates of unemployment and under-employment (Association of Graduate Careers Advisory Services [AGCAS] Disability Task Group, 2021).

Evidently, there are multiple reasons why the transition from university may be challenging, but studies examining the experiences of autistic graduates are necessary to better understand the transition and support needed. To date, there is a concerning paucity of research on this topic (Cashin, 2018). Vincent (2019) interviewed 21 autistic students and graduates about their perception of the transition out of university. Vincent (2019) found that the transition could be a source of anxiety, especially for those who did not have a clear plan for their next steps, and one coping mechanism was avoiding engaging with the transition process. Some participants reported feelings of loss, for example in terms of independence, friendships, and momentum. However, others, often those with a clear plan, viewed the transition as a positive departure, with optimism about their future. For these participants, the transition was considered a shift into adulthood and associated with positive identity development.

Thus, Vincent (2019) provides qualitative insight into both the practical and psychological phenomena associated with the transition from university and highlights the importance of transition planning. Another qualitative study by Vincent and Fabri (2020) noted the role of the ecosystem around autistic students entering employment, such as their family, university support services, and their intended industry and employer. Their study highlighted the importance of a supportive network across this ecosystem, as well as the need for societal level appreciation of autistic people. While these studies help to inform the *focus* of and *need* for support, it is also important to consider in more detail the *format* of potential support systems. Insight may be provided by examining the effectiveness of current support for the transition into and during university.

### University Support

Support designed for the transition *into* university is underpinned by evidence that this is a period of increased vulnerability for autistic people (Adreon and Durocher, 2007; Chown and Beavan, 2010; Beresford et al., 2013; Mitchell and Beresford, 2014; Van Hees et al., 2015; Elias and White, 2018). Such support often takes the form of pre-university summer programs, which provide direct experience of university life. Participant feedback on such programs is positive, with attendees reporting a reduction in concerns and an increase in optimism (Lei et al., 2019).

Once at university, support can be substantial and wide ranging, including group (e.g., social clubs and peer support groups), as well as one-to-one support (e.g., mentoring; Anderson et al., 2017). Both peer and specialist mentoring (involving a one-to-one relationship between a professional mentor and student mentee) can effectively support autistic students with their academic and mental health needs during university (Knott and Taylor, 2014; Ames et al., 2015; Hillier et al., 2019; Duerksen et al., 2021), especially when there is a personalized approach underpinned by a strong mentor-mentee partnership (Roberts and Birmingham, 2017; Siew et al., 2017; Lucas and James, 2018; Thompson et al., 2018; Thompson et al., 2020).

Thus, there is preliminary evidence that *pre*-university transition planning, support groups and mentoring can effectively support autistic students’ into and during their time at university. However, these forms of support do not specifically focus on preparing students for the transition *out* of university, and typically do not report on outcomes related to preparing students for independence (Flegenheimer and Scherf, 2021). With the transition *into* university the destination is known, and certain experiences predicted, thus it can be planned for. With the transition *out of* university there are more unknown variables, meaning the need for support may be even greater. Additionally, the degree of support an autistic person may experience during their transition into university and across their studies could mean that an absence of support for the transition out would be keenly felt.

Entering employment can present autistic people with both challenges and opportunities, but the right support can make all the difference (Hedley et al., 2018). To date, few studies have explicitly examined autistic perspectives on factors facilitating the university to work transition and the support universities provide to help prepare students for employment. Pesonen et al. (2020) interviewed 17 current autistic students, two autistic students who had not completed their studies, and 13 autistic graduates, from Finland, France, Netherlands, and United Kingdom. Careers support and internships were considered valuable, but barriers to access were identified such as the need to be self-directive. Support was considered most beneficial when individualized and provided in a caring manner. This study provides useful preliminary insight into autistic individuals’ perspectives; however, it can be challenging to generalize the results due to the diverse students’ statuses, e.g., students versus graduates and their range of country of study. Another study by Pesonen et al. (2021) with professionals (such as career advisors, academic tutors, employers) qualitatively analyzed suggested support strategies for autistic students seeking employment. The professionals suggested taking a person-centered, strengths-based approach, with a need for autism acceptance. Since the pre-existing studies have been qualitative, further quantitative data would be beneficial to further understand graduate’s experiences, and further understand what supports autistic students’ desire and need for the transition out of university, and whether any existing provision is well-suited to meet their needs.

### Study Rationale and Aims

Given the poorer graduate employment outcomes of disabled students, the latest Association of Graduate Careers Advisory Services [AGCAS] Disability Task Group (2021) report calls for further research on the barriers and facilitators to disabled graduates achieving their career ambitions. Furthermore, transitional and vocational issues have been identified as a priority for autistic adults specifically (Nicholas et al., 2017), although there is a lack of research on this topic (Cashin, 2018). It is important to understand what aspects of the transition autistic graduates find difficult, and why. Further, increased understanding of the types of support accessed or desired is needed, as well as what the impact of such support could be. The current explorative study therefore aimed to investigate the experiences of the transition out of university for autistic students. Both barriers and facilitators were examined, with consideration of both emotional support and career-focused support.

We used an in-depth mixed-methods survey with both qualitative and quantitative questions, to ensure that the study not only gathered numerical data but also heard the perspectives of our participants. Taking a mixed-methods approach can avoid a mismatch between the desired focus of support and support provision, which can frequently occur for disabled students (Anderson et al., 2017). An online survey was selected to best reach a range of participants; the study sample comprised autistic adults who had recently graduated from United Kingdom institutions, to capture the experiences and perceptions of people who had recently experienced the transition (rather than students approaching the transition). Content analysis was used to analyze the open data; this was chosen as the most appropriate analytic technique both as the study was conducted from a broadly realist research lens, believing that useful insights can directly be gained from what autistic graduates have to say about their experiences, and as it is suitable for the quantity of qualitative data generated within open survey items. The knowledge acquired from this research could serve to increase understanding of the transition out of university for autistic students and inform support provision.

## Materials and Methods

### Participants

Thirty-four autistic graduates (26 female, seven male, one preferred not to say) with a mean age of 27.78 (*SD* = 5.52, range 21–44) participated. The majority (*n* = 29) had studied full-time, three part-time, and two both full and part-time. Seven had graduated in 2018, eight in 2017, three in 2016, five in 2015, four in 2014, and seven in 2013. In line with national statistics (Higher Education Statistics Agency [HESA], 2021), most graduated with an upper second-class honors (*n* = 18), 12 with a first, three with lower second-class honors, and one a degree without honors. Participants had graduated from 26 different universities with 18 studying Science, Technology, Engineering, and Mathematics subjects and 16 Arts and Humanities.

Between November 2018 and February 2019, participants were recruited using convenience and voluntary sampling. Those who had graduated within the last 5 years from the authors’ universities and had declared an autism diagnosis were invited to participate via an email from their University Disability Service which included the information sheet and study link. In addition, the study was advertised on Twitter, and interested participants were invited to contact the research team for further information. The study was conducted in accordance with the British Psychological Society guidelines for ethical practice, and ethical approval was granted by all authors’ institutions. Informed consent was obtained from all participants, and debriefing information was provided at the end.

All participants reported a diagnosis of an Autism Spectrum Condition. Most had been diagnosed between the ages of 18–25 (*n* = 13), followed by those who had been diagnosed between the ages of 12 and 17 (*n* = 9) and over the age of 26 (*n* = 8). Some participants had been diagnosed under the age of 11 (*n* = 4). Qualitative responses indicated that some participants received their diagnosis after completing university; these participants were retained in the sample to include the experiences of autistic individuals who receive a late diagnosis. All participants scored above 14 on the Ritvo Autism and Asperger Diagnostic Scale-14 (RAADS-14; Eriksson et al., 2013; mean = 31.38; *SD* = 6.55; range = 19–41). Many participants reported a co-occurring mental health condition: specifically, 18 reported anxiety, 15 reported depression, 10 participants reported another mental health condition, most frequently PTSD (*n* = 4), and 10 reported other neurodevelopmental conditions, most commonly a specific learning difference of dyslexia, dyspraxia, and/or AD(H)D (*n* = 5).

### Materials and Procedure

Participants completed an online survey, constructed using Qualtrics (Qualtrics, Provo, UT, United States) survey software. The measures included are outlined below in the order presented. The survey took around 25 min and participants received a £5 gift voucher.

#### Participant Characteristics

Participants were asked about their terminology preferences and this language was used throughout; most preferred “autistic students” (*n* = 28) and six preferred “students with autism.” Demographic items included sex, age, diagnoses, and age of diagnoses, followed by information on undergraduate degree topic, year started, graduation year, and degree classification. Participants then completed a measure of wellbeing–the Warwick-Edinburgh Mental Well-Being Scale (WEMWBS; Tennant et al., 2007), which includes 14 items covering feeling and functioning aspects of mental wellbeing over the last 2 weeks, with items answered a five-point Likert scale. Responses are summed to create a total score, ranging from 14 to 70, with higher scores indicating greater well-being. Next, they completed questions about autistic characteristics, from the RAADS-14 (Eriksson et al., 2013). This measure contains 14 statements covering aspects such as mentalizing, social anxiety, and sensory reactivity answered on a four-point Likert scale.

#### The Transition Out of University

Participants rated the extent to which they had felt seven different emotions, including sadness, acceptance and calm, selected from Plutchiks’ (1991) theory of emotion, in relation to the transition. They also answered how prepared they had felt for the transition. All items were rated using a five-point Likert scale [“strongly disagree” (1) to “strongly agree” (5)]. Strongly agree and agree responses were combined to determine the percentage who reported feeling each emotion overall.

Transition support questions covered support received in preparation for the transition out of university. Participants were asked “While studying for your undergraduate degree, how supported did you feel by the university in terms of preparing for the transition out of university?” and answered using a five-point scale [“not very well supported” (1) to “very well supported” (5)]. They were also asked “During the last 6 months of your undergraduate degree did you access any emotional support related to the transition out of university?” and responded “yes” or “no” to options from a list (e.g., specialist mentor, personal tutor). Each form of support accessed was rated on a five-point scale [“not very helpful” (1) to “very helpful” (5)]. We also asked participants two open questions: “In what ways could the support you received for the transition out of university be improved?” and “What other support would you like your university to have offered for the transition out of university?” Then, participants selected which transition-related support they had accessed since graduating from a list and rated their helpfulness, as above.

#### Post-graduation Plans

We asked participants about their plans post-graduation, including what they were currently doing and what career-related support they had received. We asked participants who had graduated in 2018 to indicate what their main plan was for the next 6 months, and whether they planned to do anything else in the next 6 months, alongside their main plan. Options included “Paid employment,” “Voluntary work or an internship,” “Post-graduate study,” “Gap year or traveling,” “Don’t know yet,” and “Other, (please specify).” For the first three options, we asked whether a position had yet been secured (“yes,” “no,” or “awaiting outcome of application(s)”). Participants who graduated 1–5 years ago indicated what they had done in the first 6 months after graduation, as well as what they were currently doing in terms of work or further study.

All participants who had secured paid employment were asked how their job related to their undergraduate degree with response options of “It requires a university degree, and is related to the subject of my degree,” “It requires a university degree, and is not related to the subject of my degree,” “It does not require a university degree, and is related to the subject of my degree,” or “It does not require a university degree, and is not related to the subject of my degree.”

Regarding career support, participants rated the question “While studying for your undergraduate degree, how supported did you feel by the university in terms of preparing for your future career?” using the five-point scale detailed above. We also asked whether, during the last 6 months of their undergraduate degree, participants had accessed any career-focused support from a list and rated their helpfulness as above. We then asked two further open questions: “In what ways could the support you received for careers be improved?” and “What other support would you like your university to have offered for careers?” Additionally, participants were asked which forms of career support they had accessed since from a list and rated their helpfulness as before.

The survey concluded with a “final thoughts” section with two open questions: “Is there anything else you would like to say about your experience of the transition out of university?” and “What advice would you give to autistic students who are in their final year at university, to help them plan for when they finish university?”

### Design and Analysis

A mixed-methods exploratory design was used within a broadly realist research lens; we believed that our participants would self-report useful insights on the topic within both closed and open items in an online survey. Descriptive statistics are reported for the quantitative questionnaire items. Open-ended questions were analyzed using conventional data-driven content analysis (Hsieh and Shannon, 2005) to identify categories of answers.

Content analysis involves categorizing open data, enabling descriptive quantitative information to be reported about the extent to which different aspects are reported by participants. Data-driven content analysis involves developing a coding scheme based upon what is said within the data, rather than interpreting answers through a pre-existing framework derived from existing literature; this approach was chosen to best capture what autistic graduates reported about their support experiences. Developing the coding scheme necessarily involves some degree of researcher interpretation of the data. However, compared to some qualitative analysis techniques, content analysis involves less researcher interpretation as the focus is upon identifying and categorizing what is said by participants.

One author (AJ) initially read all the participants’ responses and generated an initial coding scheme with categories and subcategories which most fully captured the different aspects reported by participants. The coding scheme included brief descriptions of all categories and subcategories for each open question, with examples of the type of open responses which would fit within each category/sub-category. This coding scheme was then checked and refined with the other two authors to promote analytic rigor, which involved them reading over the qualitative data and considering whether any aspects of their answers would have been omitted or obscured by the proposed coding scheme. Following the agreement of the coding scheme, all responses were coded into the categories and sub-categories by AJ. This involved reading each open answer and selecting each category and sub-category which represented it; answers could be coded in multiple categories and sub-categories to most fully capture what participants said.

Coding was then further checked by the other two authors which involved them reviewing which categories and sub-categories each open answer had been coded into; a small number of cases where queries were raised over the most appropriate coding were fully considered and resolved in line with the coding framework. Due to high overlap in the categories identified for the two open questions about improvements to current support and desired support (for both careers and the transition more generally), we combined coding across these two questions. Two participants did not provide answers to any of the open questions; all other participants answered two or more open questions. Percentages reported for categories and sub-categories are out of the total sample of 34 participants, including those who did not give an answer for that question. Brief descriptions of each main category and a representative quotation are included within the tables reporting the content analysis findings.

## Results

### The Transition Out of University: Emotions

Nearly 80% of participants reported having felt fear toward the transition out of university, with 53% having felt sadness (Table 1). Less than a third had felt calm about or prepared for the transition. Over half had felt acceptance and anticipation, with few expressions of anger. Additionally, the mean wellbeing (WEMWBS) score for our participants was 41.91 (*SD* = 9.97; range = 20–66). Population surveys (e.g., Braunholtz et al., 2007) indicate that the mean score of the general public is 51, and scores one standard deviation below this (<42.5) indicates low well-being.

**TABLE 1:** Participants’ emotions regarding the transition out of university (rated from 1 to 7).

	Fear	Acceptance	Anticipation	Sadness	Joy	Calm	Prepared	Anger
Mean (*SD*)	3.91 (0.83)	3.56 (1.02)	3.50 (0.90)	3.50 (0.90)	3.00 (0.83)	2.74 (1.02)	2.62 (1.05)	1.88 (1.01)
% Experienced	79.41	58.82	64.71	52.94	38.24	32.35	26.47	8.82

### The Transition Out of University: Emotional Support

Overall, participants were somewhat negative regarding how well supported they felt for the transition out of university, with a mean score of 2.12 (*SD* = 0.98), equating to “disagree.” In the 6 months prior to graduation, 58.82% had accessed emotional support for the transition. Over one-third had sought emotion-related support from a specialist autism mentor, with around a fifth having spoken to either a disability advisor or their personal tutor. Less than 10% had spoken to a wellbeing officer or counselor. Support from all sources was generally rated as helpful (Table 2).

**TABLE 2:** Participants’ use and perception of emotion-related transition support and career-related transition support, pre- and post-graduation.

	Emotion-related support				Career-related support			
	Pre-graduation		Post-graduation		Pre-graduation		Post-graduation	
Source of support	*n* (%) utilized	Helpfulness Mean (SD)	*n* (%) utilized	Helpfulness Mean (SD)	*n* (%) utilized	Helpfulness Mean (SD)	*n* (%) utilized	Helpfulness Mean (SD)
Specialist autism mentor	12 (35.3)	3.83 (1.40)	6 (17.6)	4.17 (1.17)	9 (26.5)	3.89 (1.70)	5 (14.7)	3.60 (1.14)
Disability advisor	8 (23.5)	3.13 (1.64)	7 (20.6)	3.14 (1.77)	8 (23.5)	3.63 (1.69)	–	–
Wellbeing or welfare officer/counselor/therapist	6 (17.6)	4.00 (0.89)	2 (5.88)	5.00 (–)	–	–	–	–
Undergraduate personal tutor	7 (20.6)	4.00 (0.82)	5 (14.7)	3.80 (1.64)	17 (50.0)	3.71 (1.36)	3 (8.82)	3.67 (2.31)
Post-graduate personal tutor	–	–	6 (17.6)	2.33 (1.37)	–	–	6 (17.6)	3.67 (1.75)
Workplace/volunteer placement mentor	–	–	3 (8.82)	3.33 (0.58)	1 (2.94)	5.00 (–)	5 (14.7)	2.80 (1.79)
University careers service	–	–	7 (20.6)	2.71 (1.38)	16 (47.1)	2.69 (1.14)	6 (17.6)	3.17 (1.33)
National careers service	–	–	–	–	–	–	3 (8.82)	3.67 (1.53)
Other–recruitment agency	–	–	–	–	–	–	1 (2.94)	4.00 (–)

*– represents a lack of data, for example because n = 1 (thus no SD possible), helpfulness was not rated, or the source of support was not specified/available pre/post-graduation.*

Post-graduation, 44.12% of participants had accessed emotion-related transition support. Similar sources of support were utilized post-graduation as pre-graduation and were generally considered to be helpful. In addition, participants had consulted their post-graduate tutor, workplace mentor, and University’s Careers Service. However, these were rated as neutral or not helpful (Table 2). Overall, 67.65% of participants accessed emotion-related transition support either pre- or post-graduation.

Content analysis of qualitative answers to questions about potential improvements and desired transition support indicated that just under one-third of participants would have valued support preparing for the life changes, both in terms of the emotional aspect and adjusting to non-university life (Table 3). Many answers referenced careers and employment support. Nearly a third of participants desired support with employment access, just over a quarter discussed career support, specifically reporting a need for more career guidance, help finding employment and making contacts. Some wanted support with post-graduate study, such as help with understanding options and applications, and with accessibility. Other answers concerned the nature and timing of support, desiring support that was earlier, for not just when in crisis, and more positive support. Two participants felt that no extra support was needed, and nearly a quarter said they had received little or no transition support, either out of choice or lack of access.

**TABLE 3:** Content analysis of participants’ suggestions for transition support additions or improvements, showing categories (in bold) and sub-categories (bullet pointed).

Categories and sub-categories, with illustrative quotations	%	*N*
**Preparation for life changes**–transition support could be improved through greater support around preparing for the life changes involved in leaving university “*A bridging course would have been helpful and discussion about what would happen. For me it all seemed to end very suddenly as I didn’t really think about it too much and I wasn’t prepared*”	**32.35**	**11**
•Emotional support	14.71	5
•Support adjusting to non-university life	17.65	6
**Employment access**–transition support could be improved through greater support around access to employment “*It would have been nice if there had been some advice for people with disabilities. For example, tips for accessing the workplace, tips for overcoming the additional challenges people face during interviews, and so on*”	**29.41**	**10**
•Accessibility and reasonable adjustments	11.76	4
•Understanding working life	8.82	3
•Applications and interviewing support	8.82	3
•Disability positive employers	2.94	1
**Careers support**–transition support could be improved through greater careers support “*Help with careers so [I] could find next steps*”	**26.47**	**9**
•Careers guidance	14.71	5
•Support finding employment	8.82	3
•Careers contacts	5.88	2
**Post-graduate study support**–transition support could be improved through greater support around post-graduate study “*Offer more encouragement or accessibility if possible to places or returning to the university*”	**17.65**	**6**
•Options and applications	8.82	3
•Accessibility and reasonable adjustments	5.88	2
•Preparation for the transition	2.94	1
**Nature of Support**–the nature of the transition support available could be improved “*I could have known if there were any services around, it could have been made more clear. There was mentoring but it would have been helpful if I could have had specific help on Post-University life*”	**17.65**	**6**
Not just when in crisis	8.82	3
More positive support	5.88	2
Make it easier to access wider support	2.94	1
**Timing of Support**–the timing of transition support could be improved “*Slowly building up to leaving over the last year- maybe a timeline, things to think about, when to do what etc*.”	**14.71**	**5**
•Earlier support	11.76	4
•Recognition of symptoms	2.94	1
**No Changes Needed**–“*None. I was quite happy*.”	**5.88**	**2**
**Little or No Support Received**–“*I received no specific support regarding my transition out of university so anything would be an improvement*”	**23.53**	**8**
•No Support Available	11.76	4
•Challenges Accessing Support	5.88	2
•Chose not to Access Support	5.88	2
**Unclear Answer**	**2.94**	**1**
**No Answer**	**11.76**	**4**

*Sub-category n can total more than the category n as responses could be coded under multiple sub-categories.*

### Post-graduation: Future Plans

Seven participants had graduated within the last 6 months. One indicated that they did not have a post-graduation plan, while six reported plans and activities. Two of these six were pursuing paid employment (one while also caring for a family member) and two had secured a place on a post-graduate course (one was also considering part-time employment). Another was on a health-orientated gap year (while also hoping to pursue post-graduate study), and one was self-studying learning a language. Three of these six participants had additionally secured voluntary work or an internship, while the other three intended to do so.

Of the 27 participants who graduated 1–5 years ago, 88.89% had been in post-graduate study and/or paid employment in the first 6 months following graduation (Table 4). However, only four of the 11 employed participants had secured a role that required a university degree, with three being related to their degree subject. The remaining seven had a role that did not require a university degree, six of whose job was not related to their degree subject.

**TABLE 4:** Pursuits of participants who graduated 1–5 years ago.

	First 6 months post-graduation *n* (%)	Now
		*n* (%)
Post-graduate study only	11 (40.7)	8 (29.6)
Post-graduate study + paid employment	2 (7.41)	3 (11.1)
Post-graduate study + volunteering/internship	2 (7.41)	2 (7.41)
Paid employment only	8 (29.6)	9 (33.3)
Paid employment + volunteering/internship	1 (3.70)	0
Unemployed	2 (7.41)	3 (11.1)
Volunteering/internship only/ + gap year	1 (3.70)	2 (7.41)

For those who had graduated 1–5 years prior, trends were similar; 81.48% were undertaking post-graduate employment and/or studying. However, only four of the 12 employed participants had secured a role that required a university degree, with three being related to their degree subject. The remaining eight had a role that did not require a university degree, six of whose job was not related to their degree subject.

### The Transition Out of University: Career Support

Participants were neutral regarding how well supported they felt in terms of preparing for their future career with a mean score of 2.39 (*SD* = 1.20). In the 6 months prior to graduation, 76.47% had accessed career-related support (Table 2). Around 50% had spoken with their undergraduate tutor or utilized their undergraduate degree institution’s Careers Service. Around 25% had sought career-related support from a disability advisor or specialist mentor. Support was considered helpful, with the exception of the Careers Service which received a neutral mean helpfulness rating of 2.69 (*SD* = 1.14).

Post-graduation, 38.24% of participants had accessed career-related support, only one of whom had not accessed careers support prior to the transition. Thus, 20.59% of participants had not accessed career support either before or after the transition. Furthermore, 17.65% did not access either career or emotional support, pre- or post-graduation.

Of those who did access career-related support post-graduation, around 15% had sought support from their university’s Careers Service, post-graduate tutor, specialist mentor, or workplace mentor (Table 2). Less than 10% of participants sought support from their undergraduate tutor or the National Careers Service. All forms of support were rated between 2.8 and 4 for helpfulness (i.e., neutral to helpful).

Content analysis of answers to questions about potential improvements or desired career-related support (Table 5) identified that over a quarter felt that autism-focused support would be beneficial, specifically in terms of greater understanding of autistic students’ needs and more accessible support. Nearly a quarter indicated a need for more career planning support, and a fifth desired employment access support, including identifying disability positive employers, applications or interviewing support, and accessibility/adjustments. Others suggested careers connections support and two mentioned post-graduate study support. Four felt no extra support or improvements were needed, and around one-fifth had received little or no support either out of choice or lack of access.

**TABLE 5:** Content analysis of participants’ suggestions for career-related support additions or improvements, showing categories (in bold) and sub-categories (bullet pointed).

Categories and sub-categories, with illustrative quotations	%	*N*
**Autism Focused Support**–careers support tailored to autistic students would be helpful “*Autistic-specific career workshops or workshops in smaller groups*”	**26.47**	**9**
•More understanding of autistic students’ needs	17.65	6
•Autism specific careers support	8.82	3
•More accessible careers support	8.82	3
**Career Planning Support**–more careers planning support would be helpful “*More clarity about my career paths with the course I was doing*”	**23.53**	**8**
•Awareness of possible career paths	11.76	4
•Awareness of wider career options	11.76	4
•Making plans for the future	2.94	1
**Employment Access**–careers support could be improved through greater support around access to employment “*It would have been good to have gained some advice on career fields that are Autism/disability-friendly*”	**20.59**	**7**
•Disability positive employers/fields	8.82	3
•Interviewing support	8.82	3
•Accessibility and reasonable adjustments	5.88	2
**Career Connections**–careers support could be improved through greater support in making contacts and finding opportunities “*Careers [support] would be good if I could get a list of contacts for possible employers. Inviting more companies to the campus that operate in different fields*”	**17.65**	**6**
•Finding employment	8.82	3
•Careers contacts	5.88	2
•Campus work opportunities	5.88	2
**Nature of Support**–the nature of the careers support available could be improved “*Understanding of autism by career services. I found there was an expectation to fail and just being told to take a year to adjust and then try for jobs whereas I just wanted to get on with work and found this bad advice*”	**17.65**	**6**
•More positive support	8.82	3
•More advanced support	8.82	3
**Post-graduate Study Support**–careers support could be improved through greater support around post-graduate study “…*a list of supportive universities and contact with services at those universities about the support they could put in place if I was to come and that to actually be carried through*”	**5.88**	**2**
•General information	5.88	2
•Accessibility and reasonable adjustments	2.94	1
**No Extra Support or Improvements Needed**–“*I was happy with the support I received*”	**11.76**	**4**
**Little or No Support Received**–“*I chose not to seek out most support for post-university life so I can’t really offer any improvements*”	**20.59**	**7**
•Chose not to access support	11.76	4
•No support available	5.88	2
•Unclear why little support received	2.94	1
**No Answer**	**17.65**	**6**

*Sub-category n can total more than the category n as responses could be coded under multiple sub-categories.*

### Final Thoughts on the Transition

When asked whether there was anything else they would like to say about their transition out of university (Table 6), a third of participants commented upon their university experience with most making negative evaluations. A fifth referenced post-graduate study, with most reporting having had a positive transition experience and some having concerns about leaving their post-graduate degree. Around a sixth made evaluative comments about the transition, reporting both positive and negative experiences, and some referenced worries about their future.

**TABLE 6:** Content analysis of participants’ additional open comments about their transition out of university, showing categories (in bold) and sub-categories (bullet pointed).

Categories and sub-categories, with illustrative quotations	%	*N*
**University Experience**–participants’ experiences of university or of support for the transition out of university “*[it] kinda sucks getting dropped at the end*”	**32.35**	**11**
• Positive evaluation of university experience	5.88	2
• Negative evaluation of university experience	8.82	3
• Negative evaluation of university transition support	14.71	5
• Difficulties due to diagnosis after graduation	5.88	2
**Post-graduate Study**–answers related to the transition into post-graduate study or the eventual transition out of post-graduate study “*I returned, so I feel like I haven’t exactly transitioned out of university. I am currently in my final year of Ph.D. and am somewhat uncertain as to what will happen afterward as even now I feel I am going to have difficulty finding employment at the end*”	**20.59**	**7**
•Positive feelings about transition into post-graduate study	14.71	5
•Concerns and difficulties about transition into post-graduate study	5.88	2
•Worries about leaving post-graduate study	11.76	4
**Transition Out of University**–participants’ experiences of the transition out of university “*Still 3 years after graduation I feel like I am not properly equipped for this adult life. I don’t feel like anybody expected me to be and feeling like there was little belief in me having any future prospects still makes me feel sad and uncertain. I’m faced with years of life that I do not know how to fill*”	**17.65**	**6**
•Positive evaluation of the transition	8.82	3
•Negative evaluation of the transition	11.76	4
•Valued social support	2.94	1
**Worries About the Future**–participants’ current worries about the future “*Despite feeling ready to graduate, I still find the idea of not going back to the university, being in lectures, or seeing lecturers again distressing as I became so used to that routine*”	**11.76**	**4**
•Going into/finding employment	8.82	3
•Housing post-university	2.94	1
•Loss of university routine	2.94	1
•Friendships post-university	2.94	1
**Not Sure/Answer Unclear**	**14.71**	**5**
**No Answer**	**26.47**	**9**

*Sub-category n can total more than the category n as responses could be coded under multiple sub-categories.*

When asked what advice they would give to autistic students in their final year (Table 7), just over two-fifths of participants gave advice centered on forward planning with nearly a third advising students to plan early, and others suggesting they should do their research but aim to balance studying and career planning. A fifth advised students to prepare for the upcoming life changes both in terms of what their emotional and practical needs would be, and around one-sixth gave advice regarding managing expectations for the future. Answers concerning careers and employment included preparation for the workplace, particularly around possible adjustments, several avenues of careers preparation, and building experience while at university. Other advice concerned making use of university support, either before or after graduation.

**TABLE 7:** Content analysis of participants’ advice for final year autistic students, showing categories (in bold) and sub-categories (bullet pointed).

Categories and sub-categories, with illustrative quotations	%	*N*
**Planning**–advice which related to planning for the future “*Start thinking about your plan well in advance*”	**41.18**	**14**
•Plan early	29.41	10
•Do your research	11.76	4
•Balance studies and career planning	5.88	2
**Prepare for Life Changes**–preparing for the life changes involved in the transition out of university, in terms of either your practical or emotional needs “*Try to plan ahead for the changes to your environment and your support network as that will help relieve some anxiety over the unknown*”	**20.59**	**7**
•Practical needs	14.71	5
•Emotional needs	14.71	5
**Expectations for the Future**–advice about the expectations students should have for the transition “*The world doesn’t end but it’s not easy*”	**17.65**	**6**
•Expect transition to be difficult	11.76	4
•Don’t worry about the future	5.88	2
**Prepare for Adjustment to Workplace**–consider in advance the need to adjust to the workplace after university “*Find out as much information as possible about further support in the workplace*”	**17.65**	**6**
•Workplace adjustments	14.71	5
•Have an existing job	2.94	1
**Careers Preparation**–take steps to prepare for developing a career “*Try to network with people within the career you are pursuing*”	**14.71**	**5**
•Make contacts	8.82	3
•Make lots of applications	5.88	2
•Access careers support	5.88	2
**Use University Support**–make use of support available at university either during your undergraduate degree or when you enter a post-graduate degree “*Reach out and use all services available before graduating and learn whether or not you can use those resources post-graduating so there is no uncertainty*”	**14.71**	**5**
•Pre-graduation	8.82	3
•Post-graduation	8.82	3
**Build Experience**–build up experience during the degree to help with the transition and careers “*Do lots of other things at university that might improve your prospects, like volunteering*”	**11.76**	**4**
•Volunteer	5.88	2
•General	2.94	1
•Get experience in societies	2.94	1
**Not Sure**	**5.88**	**2**
**No Answer**	**8.82**	**3**

*Sub-category n can total more than the category n as responses could be coded under multiple sub-categories.*

## Discussion

Although numbers of autistic students in higher education have increased, there is little research concerning what happens when these students transition *out* of university. The current study makes an important contribution by examining the experience of autistic graduates. Emotionally, our participants reported high levels of fear and low levels of preparedness for the transition, but half also reported feeling acceptance and anticipation. Generally, participants did not feel particularly supported by their university. In the last 6 months of university, just over half had accessed emotional support for the transition, mainly from their specialist mentor, and 70% had accessed career-related support, with 50% utilizing their university’s Careers Service. Although participants rated the support they had received positively, participants expressed that they would have liked more support preparing for life after university, both emotionally and practically, greater careers advice and employment access guidance, and some desired post-graduate study support. Furthermore, participants highlighted the benefits of autism-specific support and for support to be earlier, not just at crisis points, and more positive. Of concern, open comments revealed many negative evaluations of the university experience and transition support, with some participants remaining worried about their future, however more positive experiences of post-graduate study were also reported.

Our participants expressed both negative and positive emotions related to the transition out of university. Qualitative answers also indicated a mix of positive and negative evaluations of the transition and support and ongoing worries. This finding aligns with Vincent’s (2019) interview study, where participants also reported that the transition evoked mixed emotions; a sense of anxiety and loss, that could also be accompanied by optimism and positive identity development. Thus, support for the transition out of university could not only address the students’ concerns but also encourage focus on the positives. Interestingly, our findings here are broadly comparable to the emotions reported by students with mental health conditions (Cage et al., 2021a). Our autistic participants reported many co-occurring mental health conditions, therefore the emotions experienced may be related to aspects of anxiety and depression (for example). This is in line with Accardo et al.’s (2021) study with autistic students in the United States where mental health needs were identified as a theme affecting university performance.

Around a third of participants indicated that they would have liked more support preparing for the life changes related to the transition out of university, both emotionally and practically. Graduates’ desire for support in these areas aligns with evidence on the difficulties of encountering change (Maisel et al., 2016), new physical environments and people (Van Hees et al., 2015) and on sensory challenges experienced by many autistic people (Robertson and Simmons, 2015). Support with the emotional aspects of the transition out of university is especially vital given the high co-occurrence of mental health conditions, and these findings again echo those noted for non-autistic students with mental health conditions (Cage et al., 2021a). Our findings show that it is important for universities to support autistic students with the emotional and practical aspects of transition, not just with careers and employability.

Despite over three-quarters of participants having concerns regarding the transition, only over half had accessed emotional support related to this in the 6 months prior to graduation. The most common sources of support were specialist mentors, disability advisors, and personal tutors, and the support provided was considered helpful. This is consistent with the limited extant research (Pesonen et al., 2020). However, while specialist disability professional service staff may be well equipped to support autistic students, this may not be the case for personal tutors, who tend to be members of academic staff. To ensure that students are receiving optimal guidance, additional training for personal tutors may be beneficial (Dona and Edmister, 2013) and such training is currently being developed and tested (Waisman et al., 2021). Qualitative responses did not indicate any preference for particular formats or sources of support. Preliminary investigations could focus on summer transition programs, support groups, and peer or specialist mentoring as these can effectively support autistic students with the transition into university and their time at university (Knott and Taylor, 2014; Ames et al., 2015; Roberts and Birmingham, 2017; Siew et al., 2017; Lucas and James, 2018; Thompson et al., 2018; Hillier et al., 2019; Lei et al., 2019; Thompson et al., 2020; Duerksen et al., 2021). This approach would enable support to be more autism-specific, which our participants expressed a desire for.

Fewer of our participants accessed transition-related emotional support once they had graduated, although participants who continued studying consulted their post-graduate personal tutor and institutions’ Careers Service. Qualitative comments indicated that some participants had positive experiences of the transition from undergraduate to post-graduate study, with some answers making it clear that this was a safe space which avoided the transition out of university. This finding aligns with data showing that the rate of autistic students entering post-graduate study after their first degree is higher than for non-disabled students, and for students with other types of disabilities (Association of Graduate Careers Advisory Services [AGCAS] Disability Task Group, 2021). Unfortunately, graduates may then experience the same difficulties with the transition out of this level of study, with some participants reporting worries about leaving their post-graduate course. Furthermore, employment outcomes for autistic post-graduates are concerning. Autistic post-graduate graduates on taught programs are three times more likely to be unemployed than non-disabled graduates (9.9% compared to 3.3%), while autistic post-graduate research graduates are seven times more likely to be unemployed than non-disabled graduates (16.1% compared to 2.3%; Association of Graduate Careers Advisory Services [AGCAS] Disability Task Group, 2021). Given the financial costs involved in post-graduate study, it is imperative that future research investigates how those who do enter post-graduate degrees can be better supported.

This study also examined career-related support, with our participants rating their careers service neutrally. University Careers Services are specifically designed to offer support, guidance, and opportunities pre- and post-graduation; thus, they should be the optimal form of careers support for students and recent graduates. It is therefore important to consider why it was not rated more favorably. Content analysis indicated that participants felt that it could be more tailored to autistic students’ needs, with greater understanding of autism, autism-specific support (such as workshops specifically for autistic students), and for support to be more accessible (such as in smaller groups). Some also recommended more positive support (due to negative experiences) and for support to be more detailed. The extant literature indicates that given the low numbers of disabled students (relative to the student population as a whole), Careers Service staff may have infrequent experience of supporting such students, which can result in a loss of confidence and expertise over time (Equality Change Unit, 2008). This finding indicates that regular training may be helpful for Careers Service staff. In addition Association of Graduate Careers Advisory Services [AGCAS] Disability Task Group (2021) recommends that university careers services receive appropriate resourcing in order to put effective intervention in place.

It is reassuring that most of our participants had accessed emotional or career-related support. Qualitative comments showed that some participants who received little or no support had chosen not to access it, but others perceived none to have been available or experienced challenges accessing it. This finding aligns with the perspectives of autistic students and graduates from other European universities (Pesonen et al., 2020). It is important that barriers to accessing support and potential facilitators are considered, for universities to best meet the support needs of the autistic student population. One recurring theme from our participants centered around the need for increased support in terms of accessibility and reasonable adjustments, both within the transition and in terms of future careers or post-graduate study. Our study highlights accessibility in employment and further study as a key area with which autistic students require support. Support could potentially aim to increase both students’ knowledge of accessibility and their ability to self-advocate for their rights in future work and studies. Self-advocacy has been identified as important for accessing appropriate reasonable adjustments and support during university, both for students with disabilities in general (Fossey et al., 2017) and autistic students specifically (Accardo et al., 2019). Self-advocacy may be especially important for long-term positive outcomes given the reduction in support graduates are likely to experience once they leave university.

Participants also suggested more career planning support is needed, specifically increasing students’ awareness of possible career paths related to their degrees or of career options generally. This finding aligns with data that shows a lower proportion of autistic graduates chose their current job role due to alignment with their career plan, compared with non-disabled graduates (Association of Graduate Careers Advisory Services [AGCAS] Disability Task Group, 2021). Additional support with making career connections was also desired, in line with evidence that meeting unfamiliar people can be anxiety-provoking for autistic students (Van Hees et al., 2015). Interestingly, some participants suggested that help gaining work experience on campus would especially benefit autistic students and when asked what advice they would give to final-year autistic students, one participant suggested “*use your preference for socializing with older adults to network*.” The time point for transition planning is critical; students recommended that this should begin early and not just take place at crisis points. Helping students to plan their transition out of university earlier could reduce uncertainty. Past research has indicated that programs specifically for autistic people which provide real-world work experience and vocational skills training can be beneficial for the transition to work (Flower et al., 2019).

### Limitations

This study makes an important contribution by examining autistic graduates’ experiences of the transition out of university. However, the participants in this study were self-selecting, and thus may not be representative. Recruiting a representative sample of autistic graduates can be challenging (cf. Vincent, 2019) but rather than restricting the sample to the authors’ universities, participants were recruited from throughout the United Kingdom. This resulted in participants from 26 different universities, increasing variability of experience. However, given the small number from each institution, it was not possible to examine the extent to which findings were influenced by specific university contexts. It is also important to consider the relatively small total sample size; future research could extend this exploratory research.

It is interesting to note that three-quarters of the participants were female. Although this is contrary to the traditional male:female gender distribution of 3:1 in the autistic community (Loomes et al., 2017), some studies suggest that female autistic students’ university enrollments could be as high as 47% (Dillenburger et al., 2016). Thus, our sample may be more representative of autistic people in the university community. The data was also retrospective; future research could longitudinally examine both expectations pre-transition and experiences post-transition.

We used a mixed-methods design, within a broadly realist lens, combining closed and open survey items and content analysis to most fully capture what autistic graduates had to say about careers and transition support. We note that there would be much value in further qualitative research from other philosophical positions to provide in-depth explorations of both what autistic graduates have to report and how they communicate about this topic.

### Implications

The results of this study indicate that while autistic students are accessing support, and it can be beneficial, there is potential for improvement. Specifically, universities should ensure that autistic students are supported with preparing for the life changes involved in leaving university, particularly in terms of the emotional aspects of this transition, and with the accessibility of employment and further study. Earlier planning for the transition would be beneficial, and universities should consider how to help autistic students access support earlier to facilitate this.

In addition to considering the nature of support, it is also important to consider the delivery of such support. Careers Services could become more effective by providing their staff with additional training in understanding autistic students’ needs and guidance on providing more tailored careers support. This conclusion echoes the Association of Graduate Careers Advisory Services [AGCAS] Disability Task Group (2021) report, which also recommends Careers Services provide more tailored careers support for disabled students and receive appropriate resourcing to implement interventions. Our findings also align with reported experiences of non-autistic students with mental health conditions (e.g., Cage et al., 2021a,b), suggesting that improvements are needed to support students with a range of needs more widely. Principles of Universal Design may therefore be useful when it comes to tailoring support: that support is designed with accessibility at its heart, and this serves to benefit *all* students (Gradel and Edson, 2009). Nonetheless, autism-specific understanding was clearly desired by our participants, and staff still need to better understand the unique strengths and challenges faced by autistic students.

Additionally, relationships developed through peer and specialist mentoring could be capitalized upon. Such support can effectively support autistic students with their academic and mental health needs during university (Knott and Taylor, 2014; Ames et al., 2015; Roberts and Birmingham, 2017; Siew et al., 2017; Lucas and James, 2018; Thompson et al., 2018; Hillier et al., 2019) and careers mentoring from tutors and life coaches is rated highly (Pesonen et al., 2020). Thus, peer and specialist mentoring could be extended to also help prepare students for the transition out of university. Furthermore, universities could also work with employers to reduce recruitment barriers. Supportive internships may be one viable route to providing autistic students and graduates with exposure to the work environment and the work experience that many employees require. Such internships have been rated positively by autistic graduates (Remington and Pellicano, 2019; Romualdez et al., 2020; Schall et al., 2020; Remington et al., 2021), but future initiatives should take into consideration the identified areas for improvement.

## Conclusion

The transition out of university can be a challenging time for autistic students: many autistic graduates consider transition support beneficial and recommend that it should focus on planning for life post-graduation, considering both everyday life and careers guidance. Universities should support students to access pre-existing services and support earlier transition planning. It is recommended that transition support is tailored for autistic students, while using principles of Universal Design, including ensuring that careers staff understand their needs and that support is provided on the accessibility of employment and preparing for life changes. With such support in place, a successful transition from university to post-graduation life is increasingly likely.

## Data Availability Statement

The raw data supporting the conclusions of this article will be made available by the authors, on reasonable request to the corresponding author.

## Ethics Statement

The studies involving human participants were reviewed and approved by the Department of Psychology at the University of Roehampton, Royal Holloway, and University of Reading. The participants provided their written informed consent to participate in this study.

## Author Contributions

RL and AJ conceived the research. RL, EC, and AJ managed participant recruitment and data collection, and analyzed the quantitative data. AJ took primary responsibility for the qualitative data. RL wrote the first draft of the manuscript. EC and AJ made major contributions to subsequent drafts. All authors contributed to the article and approved the submitted version.

## Conflict of Interest

The authors declare that the research was conducted in the absence of any commercial or financial relationships that could be construed as a potential conflict of interest.

## Publisher’s Note

All claims expressed in this article are solely those of the authors and do not necessarily represent those of their affiliated organizations, or those of the publisher, the editors and the reviewers. Any product that may be evaluated in this article, or claim that may be made by its manufacturer, is not guaranteed or endorsed by the publisher.

## Footnotes

1 Following the preferences of our participants, identity-first language is used in this paper.

## References

[B1] Accardo A. L. Bean K. Cook B. Gillies A. Edgington R. Kuder S. J. (2019). College access, success and equity for students on the autism spectrum. *J. Autism Dev. Disord.* 49 4877–4890. 10.1007/s10803-019-04205-8 31482372

[B2] Adreon D. Durocher J. S. (2007). Evaluating the college transition needs of individuals with high-functioning autism spectrum disorders. *Interv. Sch. Clin.* 42 271–279. 10.1177/10534512070420050201

[B3] Association of Graduate Careers Advisory Services [AGCAS] Disability Task Group (2021). *What Happens Next? A Report on the First Destinations of 2018 Disabled Graduates.* Available online at: https://www.agcas.org.uk/write/MediaUploads/Resources/Disability%20TG/AGCAS_What_Happens_Next_2021_-_February_2021.pdf (accessed July 7, 2021).

[B4] American Psychological Association [APA] (2013). *Diagnostic and Statistical Manual of Mental Disorders (DSM-V).* Arlington, VA: American Psychiatric Association.

[B5] Ames M. McMorris C. Alli L. Bebko J. (2015). Overview and evaluation of a mentorship program for university students with ASD. *Focus Autism Other Dev. Disabil.* 31 27–36. 10.1177/1088357615583465

[B6] Anderson A. Stephenson J. Carter M. (2017). A systematic literature review of the experiences and supports of students with autism spectrum disorder in post-secondary education. *Res. Autism Spect. Disord.* 39 33–53. 10.1016/j.rasd.2017.04.002

[B7] Beresford B. A. Moran N. E. Sloper T. Cusworth L. S. Mitchell W. A. Spiers G. F. (2013). *Transition to Adult Services and Adulthood for Young People with Autistic Spectrum Conditions. SPRU Working Paper*, Vol. DH 2525. New York, NY: Social Policy Research Unit.

[B8] Braunholtz S. Davidson S. Myant K. O’Connor R. (2007). *Well? What do You Think? (2006). The Third National SCOTTISH Survey of Public Attitudes to Mental Health, Mental Wellbeing and Mental Health Problems.* Edinburgh: Scottish Government Social Research.

[B9] Bury S. M. Hedley D. Uljarević M. Gal E. (2020). The autism advantage at work: a critical and systematic review of current evidence. *Res. Dev. Disabil.* 105:103750. 10.1016/j.ridd.2020.103750 32810716

[B10] Cage E. Howes J. (2020). Dropping out and moving on: a qualitative study of autistic people’s experiences of university. *Autism* 24 1664–1675. 32476451 10.1177/1362361320918750 PMC7575306

[B11] Cage E. James A. I. Newell V. Lucas R. (2021a). Expectations and experiences of the transition out of university for students with mental health conditions. *Eur. J. High. Educ.* 1–23. 10.1080/21568235.2021.1917440 [Epub ahead of print].

[B12] Cage E. Jones E. Ryan G. Hughes G. Spanner L. (2021b). Student mental health and transitions into, through and out of university: Student and staff perspectives. *J. Further High. Educ.* 45 1–13. 10.1080/0309877X.2021.1875203

[B13] Cashin A. (2018). The transition from university completion to employment for students with Autism Spectrum Disorder. *IssuesMental Health Nurs.* 39 1043–1046. 10.1080/01612840.2017.1401188 29370550

[B14] Chown N. Beavan N. (2010). *I Hope that at College I Will Have Support from Someone Who Understands What I Find difficult”: Removing Barriers to Learning for Students with Autism in Further Education. Report for Dudley College.* Dudley: Dudley college of technology.

[B15] Dillenburger K. Jordan J. McKerr L. (2016). School’s out forever: postsecondary educational trajectories of students with autism. *Austrl. Psychol.* 51 304–315. 10.1111/ap.12228

[B16] Dona J. Edmister J. H. (2013). An examination of community college faculty members’ knowledge of the Americans with Disabilities Act of 1990 at the fifteen community colleges in Mississippi. *J. Post Second. Educ. Disabil.* 14 91–103.

[B17] Duerksen K. Besney R. Ames M. McMorris C. A. (2021). Supporting autistic adults in postsecondary settings: a systematic review of peer mentorship programs. *Autism Adulth.* 3 85–99. 10.1089/aut.2020.0054 PMC8992890 36601268

[B18] Elias R. White S. W. (2018). Autism goes to college: understanding the needs of a student population on the rise. *J. Autism Dev. Disord.* 48 732–746. 10.1007/s10803-017-3075-7 28255760 PMC5581732

[B19] Equality Change Unit (2008). *Transition to Work for Disabled Students: Careers Support in Higher Education Report 2008.* Available online at: https://www.ecu.ac.uk/wp-content/uploads/external/transition-work-disabled-students.pdf (accessed November 29, 2019).

[B20] Eriksson J. Andersen L. Bejerot S. (2013). RAADS-14 screen: validity of a screening tool for autism spectrum disorder in an adult psychiatric population. *Mol. Autism* 4:49. 10.1186/2040-2392-4-49 24321513 PMC3907126

[B21] Flegenheimer C. Scherf K. S. (2021). College as a developmental context for emerging adulthood in autism: a systematic review of what we know and where we go from here. *J. Autism Dev. Disord.* 10.1007/s10803-021-05088-4 34060001 PMC8720487

[B22] Flower R. L. Hedley D. Spoor J. R. Dissanayake C. (2019). An alternative pathway to employment for autistic job-seekers: a case study of a training and assessment program targeted to autistic job candidates. *J. Vocat. Educ. Train.* 71 407–428.

[B23] Fossey E. Chaffey L. Venville A. Ennals P. Douglas J. Bigby C. (2017). Navigating the complexity of disability support in tertiary education: perspectives of students and disability service staff. *Int. J. Inclusive Educ.* 21 822–832. 10.1080/13603116.2017.1278798

[B24] Gradel K. Edson A. J. (2009). Putting universal design for learning on the higher ed agenda. *J. Educ. Technol. Syst.* 38 111–121. 10.2190/ET.38.2.d 22612255

[B25] Hedley D. Cai R. Uljarevic M. Wilmot M. Spoor J. R. Richdale A. (2018). Transition to work: perspectives from the autism spectrum. *Autism* 22 528–541. 28387577 10.1177/1362361316687697

[B26] Higher Education Statistics Agency [HESA] (2011). *Students in Higher Education Institutions: Students, Qualifiers and Staff Data Tables, Students by Disability.* Available online at: https://www.hesa.ac.uk/data-and-analysis/publications/students-2010-11 (accessed January 10, 2022).

[B27] Higher Education Statistics Agency [HESA] (2021). *Table 15 – UK Domiciled Student Enrolments by Disability and Sex 2014/15 to 2019/20..* Available online at: https://www.hesa.ac.uk/data-and-analysis/students/table-15 (accessed January 10, 2022).

[B28] Hillier A. Goldstein J. Tornatore L. Byrne E. Johnson H. (2019). Outcomes of a peer mentoring program for university students with disabilities. *Mentor. Tutor. Partner. Learn.* 27 487–508. 10.1080/13611267.2019.1675850

[B29] Hsieh H. F. Shannon S. E. (2005). Three approaches to qualitative content analysis. *Qual. Health Res.* 15 1277–1288. 10.1177/1049732305276687 16204405

[B30] Knott F. Taylor A. (2014). Life at university with Asperger syndrome: a comparison of student and staff perspectives. *Int. J. Inclusive Educ.* 18 411–426. 10.1080/13603116.2013.781236

[B31] Lei J. Calley S. Brosnan M. Ashwin C. Russell A. (2019). Evaluation of a transition to university programme for students with Autism Spectrum Disorder. *J. Autism Dev. Disord.* 50 2397–2411. 10.1007/s10803-018-3776-6 30315485 PMC7308263

[B32] Loomes R. Hull L. Mandy W. P. L. (2017). What is the male-to-female ratio in Autism Spectrum Disorder? A systematic review and meta-analysis. *J. Am. Acad. Child Adolesc. Psychiatry* 56 466–474. 10.1016/j.jaac.2017.03.013 28545751

[B33] Lucas R. James A. I. (2018). An evaluation of specialist mentoring for university students with autism spectrum disorders and mental health conditions. *J. Autism Dev. Disord.* 48 694–707. 10.1007/s10803-017-3303-1 28918532

[B34] Maisel M. E. Stephenson K. G. South M. Rodgers J. Freeston M. H. Gaigg S. B. (2016). Modeling the cognitive mechanisms linking autism symptoms and anxiety in adults. *J. Abnorm. Psychol.* 125:692. 10.1037/abn0000168 27196436

[B35] Mazefsky C. Folstein S. Lainhart J. (2008). Overrepresentation of mood and anxiety disorders in adults with autism and their first-degree relatives: what does it mean? *Autism Res.* 1 193–197. 10.1002/aur.23 19360666 PMC2939830

[B36] Mitchell W. Beresford B. (2014). Educational transitions. *Br. J. Special Educ.* 41 151–171. 10.1111/1467-8578.12064

[B37] National Educational Association of Disabled Students (2012). Working Towards a Coordinated National Approach to Services, Accommodations and Policies for Post-Secondary Students with Disabilities. Available online at: http://www.neads.ca/en/about/projects/nasp/nasp_intro.php (accessed January 10, 2022).

[B38] Nicholas D. B. Hodgetts S. Zwaigenbaum L. Smith L. E. Shattuck P. Parr J. R. (2017). Research needs and priorities for transition and employment in autism: considerations reflected in a “Special Interest Group” at the international meeting for autism research. *Autism Res.* 10 15–24. 10.1002/aur.1683 27753278

[B39] Pesonen H. Waltz M. Fabri M. Lahdelma M. Syurina E. V. (2020). Students and graduates with autism: perceptions of support when preparing for transition from university to work. *Eur. J. Special Needs Educ.* 4 1–16. 10.1080/08856257.2020.1769982

[B40] Pesonen H. Waltz M. Fabri M. Syurina E. Krückels S. Algner M. (2021). Stakeholders’ views on effective employment support strategies for autistic university students and graduates entering the world of work. *Adv. Autism* 7 16–27.

[B41] Plutchik R. (1991). *The Emotions.* Lanham, MD: University Press of America.

[B42] Remington A. Pellicano E. (2019). ‘Sometimes you just need someone to take a chance on you’: an internship programme for autistic graduates at Deutsche Bank, UK. *J. Manag. Organ.* 25 516–534. 10.1017/jmo.2018.66

[B43] Remington A. Heasman B. Romualdez A. M. Pellicano E. (2021). Experiences of autistic and non-autistic individuals participating in a corporate internship scheme. *Autism.* 26, 201–216. 10.1177/13623613211025115 34151609 PMC8750129

[B44] Richardson J. T. E. (2017). Academic attainment in students with autism spectrum disorders in distance education. *Open Learn.* 32 81–91. 10.1080/02680513.2016.1272446

[B45] Roberts N. Birmingham E. (2017). Mentoring university students with ASD: a mentee-centered approach. *J. Autism Dev. Disord.* 47 1038–1050. 10.1007/s10803-016-2997-9 28132120

[B46] Robertson A. E. Simmons D. R. (2015). The sensory experiences of adults with Autism Spectrum Disorder: a qualitative analysis. *Perception* 44 569–586. 10.1068/p7833 26422904

[B47] Romualdez A. Yirrell K. Remington A. (2020). Exploring participants’ views on a supported work internship program for autistic and learning disabled young people. *Int. J. Disabil. Manage.* 15:e3. 10.1017/idm.2020.4

[B48] Schall C. Sima A. P. Avellone L. Wehman P. McDonough J. Brown A. (2020). The effect of business internships model and employment on enhancing the independence of young adults with significant impact from autism. *Intellect. Dev. Disabil.* 58 301–313. 10.1352/1934-9556-58.4.301 32750714

[B49] Siew C. T. Mazzucchelli T. G. Rooney R. Girdler S. (2017). A specialist peer mentoring program for university students on the autism spectrum: a pilot study. *PLoS One* 12:e0180854. 10.1371/journal.pone.0180854 28704446 PMC5509180

[B50] Simonoff E. Pickles A. Charman T. Chandler S. Loucas T. Baird G. (2008). Psychiatric disorders in children with Autism Spectrum Disorders: prevalence, comorbidity, and associated factors in a population-derived sample. *J. Am. Acade. Child Adolesc. Psychiatry* 47 921–929. 10.1097/CHI.0b013e318179964f 18645422

[B51] Skokauskas N. Gallagher L. (2010). Psychosis, affective disorders and anxiety in Autistic Spectrum Disorder: prevalence and nosological considerations. *Psychopathology* 43 8–16. 10.1159/000255958 19893339

[B52] Tennant R. Hiller L. Fishwick R. Platt S. Joseph S. Weich S. (2007). The Warwick-Dinburgh mental well-being scale (WEMWBS): development and UK validation. *Health Qual. Life Outc.* 5:63. 10.1186/1477-7525-5-63 18042300 PMC2222612

[B53] Thompson C. Bölte S. Falkmer T. Girdler S. (2018). Viewpoints on how students with autism can best navigate university. *Scand. J. Occup. Ther.* 26 294–305. 10.1080/11038128.2018.1495761 30301402

[B54] Thompson C. McDonald J. Kidd T. Falkmer T. Bölte S. Girdler S. (2020). “I don’t want to be a patient”: peer mentoring partnership fosters communication for autistic university students. *Scand. J. Occup. Ther.* 27 625–640. 10.1080/11038128.2020.1738545 32180486

[B55] Van Hees V. Moyson T. Roeyers H. (2015). Higher education experiences of students with Autism Spectrum Disorder: Challenges, benefits and support needs. *J. Autism Dev. Disord.* 45 1673–1688. 10.1007/s10803-014-2324-2 25448918

[B56] van Schalkwyk G. I. Volkmar F. R. (2017). Autism Spectrum Disorders: challenges and opportunities for transition to adulthood. *Child Adolesc. Psychiatr. Clin. North Am.* 26 329–339. 10.1016/j.chc.2016.12.013 28314459

[B57] van Steensel F. Bögels S. de Bruin E. (2013). Psychiatric comorbidity in children with Autism Spectrum Disorders: a comparison with children with ADHD. *J. Child Fam. Stud.* 22 368–376. 10.1007/s10826-012-9587-z 23524401 PMC3602612

[B58] Vincent J. (2019). It’s the fear of the unknown: transition from higher education for young autistic adults. *Autism* 23 1575–1585. 10.1177/1362361318822498 30632780

[B59] Vincent J. Fabri M. (2020). The ecosystem of competitive employment for university graduates with autism. *Int. J. Disabil. Dev. Educ.* 1–17. 10.1080/1034912X.2020.1821874 [Epub ahead of print].

[B60] Waisman T. C. Cage E. Santhanam S. P. Magiati I. Dwyer P. Stockwell K. (2021). *Learning from the Experts: Evaluating a Participatory Autism and Universal Design Training for University Teaching Staff. Preprint.* Available online at: https://www.researchgate.net/publication/354431462_Learning_from_the_Experts_Evaluating_a_Participatory_Autism_and_Universal_Design_Training_for_University_Teaching_Staff (accessed January 10, 2022). 10.1177/13623613221097207 35652315

